# Impaired glucose tolerance is associated with enhanced postprandial pancreatic polypeptide secretion

**DOI:** 10.1111/1753-0407.13268

**Published:** 2022-04-19

**Authors:** Yanyun Zhao, Yue Zhou, Min Xiao, Yajing Huang, Mengmeng Qi, Zili Kong, Jingwei Chi, Kui Che, Wenshan Lv, Bingzi Dong, Yangang Wang

**Affiliations:** ^1^ Department of Endocrinology and Metabolism Affiliated Hospital of Qingdao University Qingdao China; ^2^ Medical Research Center Qingdao Key Laboratory of Thyroid Diseases Qingdao China

**Keywords:** glucose tolerance, islet function, pancreatic polypeptide, T2DM, 2型糖尿病, 糖耐量, 胰多肽, 胰岛功能

## Abstract

**Background:**

The purpose of this study is to compare serum pancreatic polypeptide (PP), insulin, C‐peptide, and glucagon in different glucose tolerance stages; analyze the influencing factors of PP secretion; and further explore the role of PP in the pathogenesis of diabetes mellitus.

**Methods:**

Data were collected from 100 subjects from hospital. According to the results of oral glucose tolerance test (OGTT), the subjects were divided into a normal glucose tolerance (NGT) group, an impaired glucose regulation (IGR) group, and a newly diagnosed type 2 diabetes mellitus (T2DM) group. PP and the related parameters were measured, and the area under the curve (AUC) 120 min after OGTT was calculated. AUC_pp_ (*AUC of PP)* was used as the dependent variable and the potentially influencing factors were used as the independent variable for multiple linear regression analysis.

**Results:**

Postprandial 60 min PP in the IGR group was higher than those in the NGT group (2973.80 [±547.49] pg·h/mL vs 2663.55 [±594.89] pg·h/mL, *p* < 0.05). AUC_pp_ was significantly higher in the IGR group (428.76 pg·h/mL, 95% confidence interval [CI] [41.06 –816.46], *p* = 0.031) and newly diagnosed T2DM group (404.35 pg·h/mL, 95% CI [5.37–803.33], *p* = 0.047) than in the NGT group. AUC_pp_ was negatively correlated with body mass index (BMI) (r = −0.235, *p* = 0.038) and positively correlated with postprandial 60 min blood glucose (r = 0.370, *p* = 0.001) and AUC_bg_ (AUC of blood glucose) (r = 0.323, *p* = 0.007). Multiple linear regression analysis indicated that there was a linear correlation between BMI, AUC_bg_, and AUC_pp_ (*p =* 0.004, *p =* 0.001), and the regression equation was calculated as: AUC_pp_ = 6592.272 + 86.275 × AUC_bg_‐95.291 × BMI (R^2^ = 12.7%, *p* < 0.05).

**Conclusions:**

Compared with NGT subjects, IGR and T2DM patients have an enhanced postprandial PP secretion. In T2DMs, the secretion of PP is mainly affected by BMI and blood glucose.

## Graphical abstract

**Highlights**

- Compared with normal glucose tolerance subjects, impaired glucose regulation and type 2 diabetes mellitus (T2DM) patients have an enhanced postprandial pancreatic polypeptide (PP) secretion.
- In patients with T2DM, the secretion of PP is mainly affected by body mass index and blood glucose.
- There may exist a potential protective effect of PP on the function of isletcells.

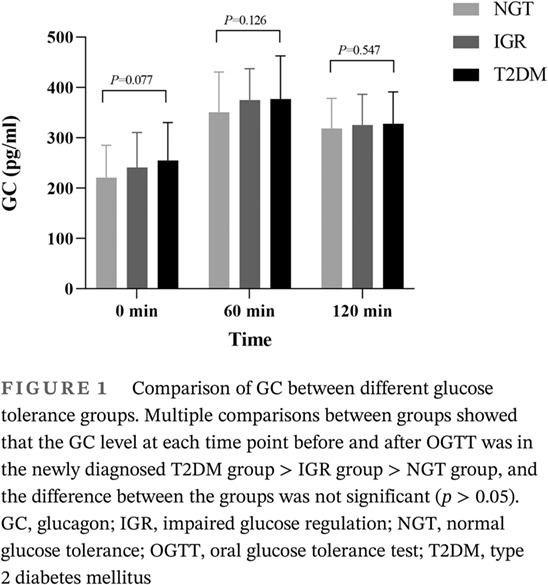

## 摘要

**背景:**

本研究旨在比较不同糖耐量阶段血清胰多肽(PP)、胰岛素、C‐肽、胰高血糖素的变化, 分析影响胰多肽分泌的因素, 进一步探讨胰多肽在糖尿病发病机制中的作用。

**方法:**

收集100例住院患者的临床资料。根据口服糖耐量试验(OGTT)结果, 将受试者分为糖耐量正常(NGT)组、糖调节受损(IGR)组和新诊断的2型糖尿病(T2D)组。测量脉压及相关指标, 计算餐后2小时曲线下面积(AUC)。以AUCpp(PP AUC)为因变量, 潜在影响因素为自变量进行多元线性回归分析。

**结果:**

IGR组餐后60 分钟PP高于NGT组(2973.80±547.49)pg·h/mLvs 2663.55[±594.89]pg·h/mL, p < 0.05)。AUCpp在IGR组(428.76 pg·h/ml, 95%可信区间[CI][41.06‐816.46], p=0.031)和初诊T2D组(404.35 pg·h/ml, 95%CI[5.37‐803.33], p=0.047)显著高于NGT组。AUCpp与体重指数(BMI)呈负相关(r=−0.235, p=0.038), 与餐后60 分钟血糖(r=0.370, p=0.001)和AUCbg(血糖AUC)呈正相关(r=0.323, p=0.007)。多元线性回归分析显示, 体重指数、AUCbg与AUCpp呈线性相关(p =0.004, p =0.001), 回归方程为:AUCpp=6592.272 + 86.275 × AUCbg‐95.291 × BMI(R2=12.7%, p < 0.05)。

**结论:**

与NGT患者相比, IGR和T2 D患者餐后PP分泌增加。在T2D中, PP的分泌主要受BMI和血糖的影响。

## 1 INTRODUCTION

There is increasing evidence demonstrating that gastrointestinal hormones not only play a central role in regulating food intake, nutrient absorption, intestinal movement, weight control, and energy balance but also in insulin secretion, glucose homeostasis, immune system regulation, etc.^1^, ^2^ With the continuous progress of people's understanding of the structure and pathophysiology of gastrointestinal hormones, gastrointestinal hormones have become a promising hot spot for the treatment of diabetes.

Pancreatic polypeptide (PP) is a gastrointestinal hormone mainly secreted by islet PP cells，which is a neuropeptide Y (NPY) family polypeptide composed of 36 amino acids.^3^, ^4^, ^5^, ^6^ Studies have shown that PP is released in a biphasic pattern after protein intake. The first secretory phase is the fast secretory phase. In this period, the circulating PP can rapidly rise to more than 4 times the basic value in a short period of time, and the peak lasts for about 30 to 60 min and is mainly regulated by vagus nerve activity.^7^ The second secretory phase, also known as the late delayed phase, can last for more than 5 h after a meal and is mainly mediated by humoral regulation and vagal nerve activity.^8^, ^9^, ^10^, ^11^ As a gastrointestinal hormone, PP can increase satiety, delay gastric emptying, inhibit gallbladder movement, and play an important role in weight loss and energy consumption.^12^, ^13^, ^14^

Evidence shows that PP can maintain sensitivity of the liver to insulin by regulating the expression of insulin receptor gene on the liver surface.^15^, ^16^ In addition, some scholars believe that PP may have an important paracrine interaction with insulin (INS) and glucagons (GCs).^17^, ^18^ The impairment of β‐cell function in chronic pancreatitis was supposed to correlate with the damage of PP cells, and PP has a potentially important impact on the survival and function of β cells.^19^, ^20^, ^21^, ^22^ All of this suggests that PP may have a potential role in the pathogenesis of type 2 diabetes mellitus (T2DM).

The research on the role of PP in the occurrence and development of T2DM is mostly limited to the early stage, and some key issues have not been fully clarified or are still controversial. This study aims to explore the secretion changes and influencing factors of PP in different stages of glucose metabolism, and further analyze the role of PP in the pathogenesis of T2DM, which will not only help to clarify the pathogenesis of diabetes but also provide new ideas for the prevention, diagnosis, and treatment of diabetes in the future.

## 2 METHODS

### 2.1 Study population

According to the 1999 World Health Organization diabetes diagnostic criteria, a total of 100 subjects, including 34 patients with impaired glucose regulation (IGR), 29 patients with T2DM, and 37 healthy subjects, were selected from the Department of Endocrinology of the Affiliated Hospital of Qingdao University and the Physical Examination Center during the same period from June 2019 to December 2019; none of the subjects had been previously diagnosed with diabetes or had received glucose‐lowering therapy. The inclusion criteria for normal glucose tolerance (NGT) group were fasting venous blood glucose (FBG) <6.1 mmol/L and 2‐hour venous BG (2hBG) <7.8 mmol/L during an oral glucose tolerance test (OGTT). IGR included impaired fasting BG (IFG) and impaired glucose tolerance (IGT), which can exist separately or simultaneously. The inclusion criterion for IFG was 6.1 mmol/L ≤ FBG<7.0 mmol/L, and the inclusion criterion for IGT was FBG<6.1 mmol/L, 7.8mmo/L ≤ 2hBG during an OGTT<11.1 mmol/L. The inclusion criterion for diabetes was FBG ≥7.0 mmol/L, or 2hBG during an OGTT ≥11.1 mmol/L, or a random venous BG ≥11.1 mmol/L with typical symptoms of diabetes.

The exclusion criteria were as follows: type 1 diabetes mellitus (T1DM), gestational diabetes, special type diabetes, secondary diabetes, and other non‐T2DM; recently taken glucocorticoid, thyroid hormone, and other drugs that can cause significantly influence on blood sugar; insulin, secretagogues, glucagon‐like peptide 1 (GLP‐1) agonist, or dipeptidyl peptidase‐IV (DPP‐4) inhibitor drugs were used in the first week of the experiment; diabetic acute complications; complicated with severe cardiovascular, liver, or kidney disease, etc; severe infection, stress, or trauma in the recent past; combined with Cushing disease, polycystic ovary syndrome, acromegaly, hyperthyroidism, and other endocrine and autoimmune system diseases; and complicated with acute or chronic pancreatitis, pancreatic malignancy, PP cell tumor, gastrointestinal ulcer, gastric cancer, liver cirrhosis, or depression and other diseases affecting PP secretion.

The protocol was designed in accordance with the Declaration of Helsinki and was approved by the ethics committee of the Affiliated Hospital of Qingdao University, and all participants provided written informed consent. The study was registered on http://www.chictr.org.cn/ under number ChiCTR2100047486.

### 2.2 Data collection

The general clinical data collection of the selected subjects included gender, age, past medical history, current medical history, smoking history, alcohol consumption history, and drug history. Clinical examinations were conducted by a trained staff group according to a standard guideline. The subjects took off their shoes, hats, and coats, and their body weight (kg), height (cm), and waist circumference (cm) were measured by a specific person under the premise of empty urine and fasting. Systolic blood pressure(SBP, mm Hg) and diastolic blood pressure(DBP, mm Hg) of the right upper limb in each subject were measured after resting for 15 min, and the mean of the two measurements was taken. The circumferential diameter of the midpoint between the costal margin and the anterior superior iliac spine was used as the measurement method of waist circumference. Body mass index (BMI) (kg/m^2^) = weight (kg) / height (m)^2^ was calculated. Through laboratory examination, we measured the following indicators: glycosylated hemoglobin (HbA1c), FBG, INS, C‐peptide (C‐P), GC, GLP‐1, urinary albumin to creatinine ratio (UACR), blood lipids (low‐density lipoprotein‐cholesterol [LDL‐c], high‐density lipoprotein‐cholesterol [HDL‐c]), free fatty acid (FFA), triglycerides (TG), total cholesterol (TC)], and liver function (alanine aminotransferase [ALT], aspartate aminotransferase [AST]), glutamyltranspeptidase, renal function (creatinine, urea nitrogen [BUN]), and uric acid.

OGTT was performed on the subjects selected from the First Outpatient Clinic and Physical Examination Center of the Affiliated Hospital of Qingdao University from June 2019 to December 2019: After the fasting blood of the subjects was extracted, 75 g glucose powder was dissolved in 250‐300 mL water, mixed well, and then the drink was finished within 10 min. According to the results of OGTT, the subjects were divided into the NGT group, the IGR group, and the newly diagnosed T2DM group. Hard physical work or extra food intake was not allowed during the experiment. The elbow venous blood was extracted at 60 min and 120 min after glucose loading to detect the BG, INS, and C‐P at each time point, and 2 mL of elbow venous blood was stored in EDTA anticoagulant tube pre‐added with DPP‐4 inhibitor and then centrifuged in batches in a laboratory centrifuge at 3000 r/min for 10 min. The upper serum was taken for the determination of PP and GC. After the collection is completed, it should be stored in a refrigerator at −80°C in the laboratory for unified determination. During the storage process, attention should be paid to avoid repeated freezing and thawing. After fasting for at least 8 h to 12 h, the blood samples were obtained at about 6:00 am the next day from the median vein of the elbow and transported to the central laboratory for detection. Siemens DCavantage glycohemoglobin analyzer was used to detect the percentage of HbA1c by monoclonal antibody agglutination method. The BG level of each specimen was detected by using the principle of glucose oxidase measurement (OLYMPUS automatic biochemical analyzer). The LDL‐C, HDL‐C, TG, TC, and FFA were determined by enzymatic method (Olympusau‐2700, Japan). The levels of INS and C‐P at each time point were determined by radioimmunoassay (XH6020γ radioimmunocounter, Xi 'an). The concentrations of PP and GC in the samples were determined by double antibody sandwich enzyme‐linked immunosorbent assay (ELISA). The detection kit was purchased from Shanghai Enzyme‐linked Biotechnology Co., Ltd.

The homeostasis model was used to evaluate insulin resistance index (homeostatic model assessment of insulin resistance [HOMA‐IR]) to assess insulin sensitivity, and the calculation formula was fasting glucose × fasting insulin / 22.5. The secretory function of islet β cells was assessed by homeostasis model (homeostatic model assessment of beta cell function [HOMA‐β]), which was calculated as 20 × fasting insulin/(fasting glucose −3.5). The area under the curve (AUC) of OGTT 120 min PP, GC, INS, C‐P, BG:

$${\mathrm{AUC}}_{\mathrm{pp}}=0.5\times {\mathrm{PP}}_{0}+{\mathrm{PP}}_{60}+0.5\times {\mathrm{PP}}_{120},{\mathrm{AUC}}_{\mathrm{gc}}=0.5\times {\mathrm{GC}}_{0}+{\mathrm{GC}}_{60}+0.5\times {\mathrm{GC}}_{120},$$

$${\mathrm{AUC}}_{\mathrm{ins}}=0.5\times {\mathrm{INS}}_{0}+{\mathrm{INS}}_{60}+0.5\times {\mathrm{INS}}_{120},{\mathrm{AUC}}_{\mathrm{cp}}=0.5\times C-P_{0}+C-P_{60}+0.5\times C-P_{120},$$

AUC_bg=_0.5 × BG_0_ + BG_60_ + 0.5 × BG_120_. Note: PP_0_, PP_60_, PP_120_, GC_0_, GC_60_, GC_120_, INS_0_, INS_60_, INS_120_, C‐P_0_, C‐P_60_, C‐P_120_, BG_0_, BG_60_, BG_120_ represent the PP, GC, INS, C‐P, and BG concentrations corresponding to fasting, 60 min, and 120 min after glucose administration, respectively.

### 2.3 Statistical analysis

SPSS software version 22.0 (SPSS, IBM Corporation, Armonk, NY, USA) was used to carry out statistical analyses. The measurement data with normal distribution were expressed as mean ± SD; those with the nonnormal distribution were expressed as the median (quartile interval); classified data were described by rate or composition ratio. The comparison of means between different glucose tolerance groups conformed to normal distribution and homogeneity of variance using analysis of variance, including single factor, multivariate and repeated measures analysis of variance. One‐way analysis of variance (ANOVA) was used for the comparison of general indexes and hormone secretion indexes of the three groups. Two‐factor repeated measure ANOVA was used for the overall analysis of PP, GC, INS, and C‐P groups and time at each time point, and multivariate ANOVA was used for pair comparison (least significant difference method was used for multiple comparisons). Pearson correlation analysis was used for normal distribution data, and Spearman correlation analysis was used for nonnormal distribution data. Multivariate stepwise regression analysis was used to build a regression model for the AUC_pp_ curve. When *p* < 0.05, the difference between groups was considered statistically significant.

## 3 RESULTS

**FIGURE 1:**
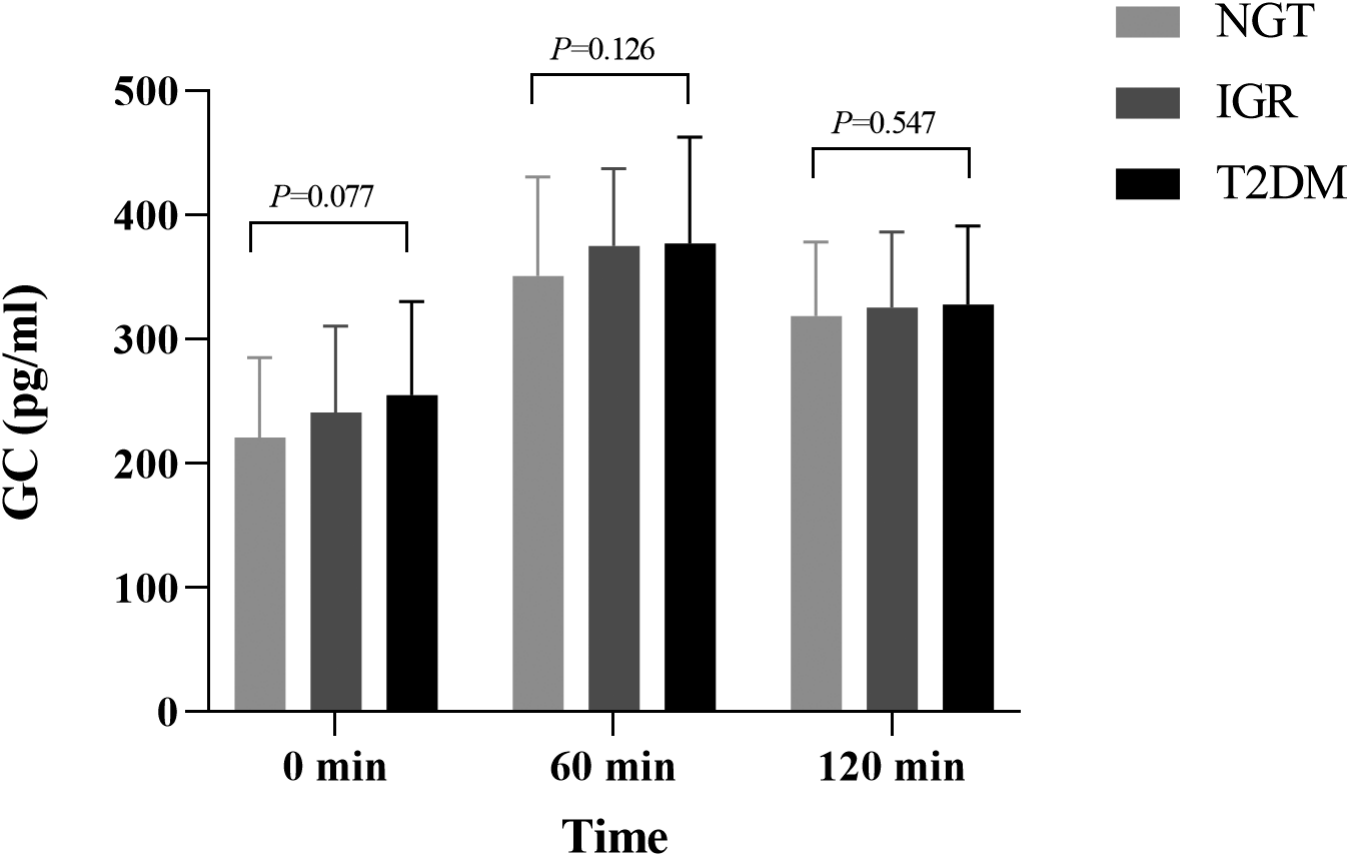
Comparison of GC between different glucose tolerance groups. Multiple comparisons between groups showed that the GC level at each time point before and after OGTT was in the newly diagnosed T2DM group > IGR group > NGT group, and the difference between the groups was not significant (*p* > 0.05). GC, glucagon; IGR, impaired glucose regulation; NGT, normal glucose tolerance; OGTT, oral glucose tolerance test; T2DM, type 2 diabetes mellitus

**FIGURE 2:**
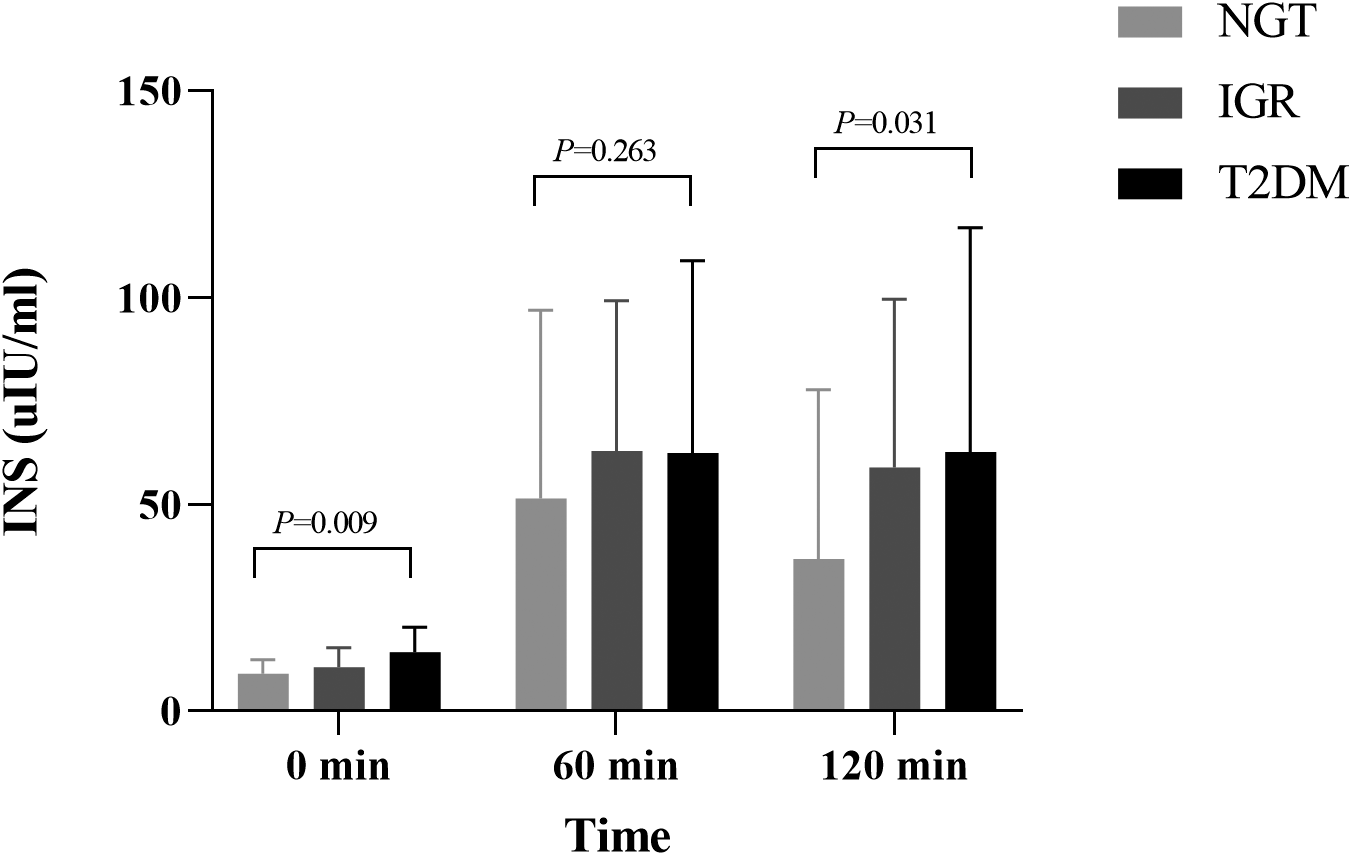
Comparison of INS between different glucose tolerance groups. Multiple comparisons among groups showed that the fasting and postglucose INS between the three groups were in the newly diagnosed T2DM group > IGRgroup > NGT group, but the difference did not reach significance (*p* > 0.05). IGR, impaired glucose regulation; INS, insulin; NGT, normal glucose tolerance; OGTT, oral glucose tolerance test; T2DM, type 2 diabetes mellitus

**FIGURE 3:**
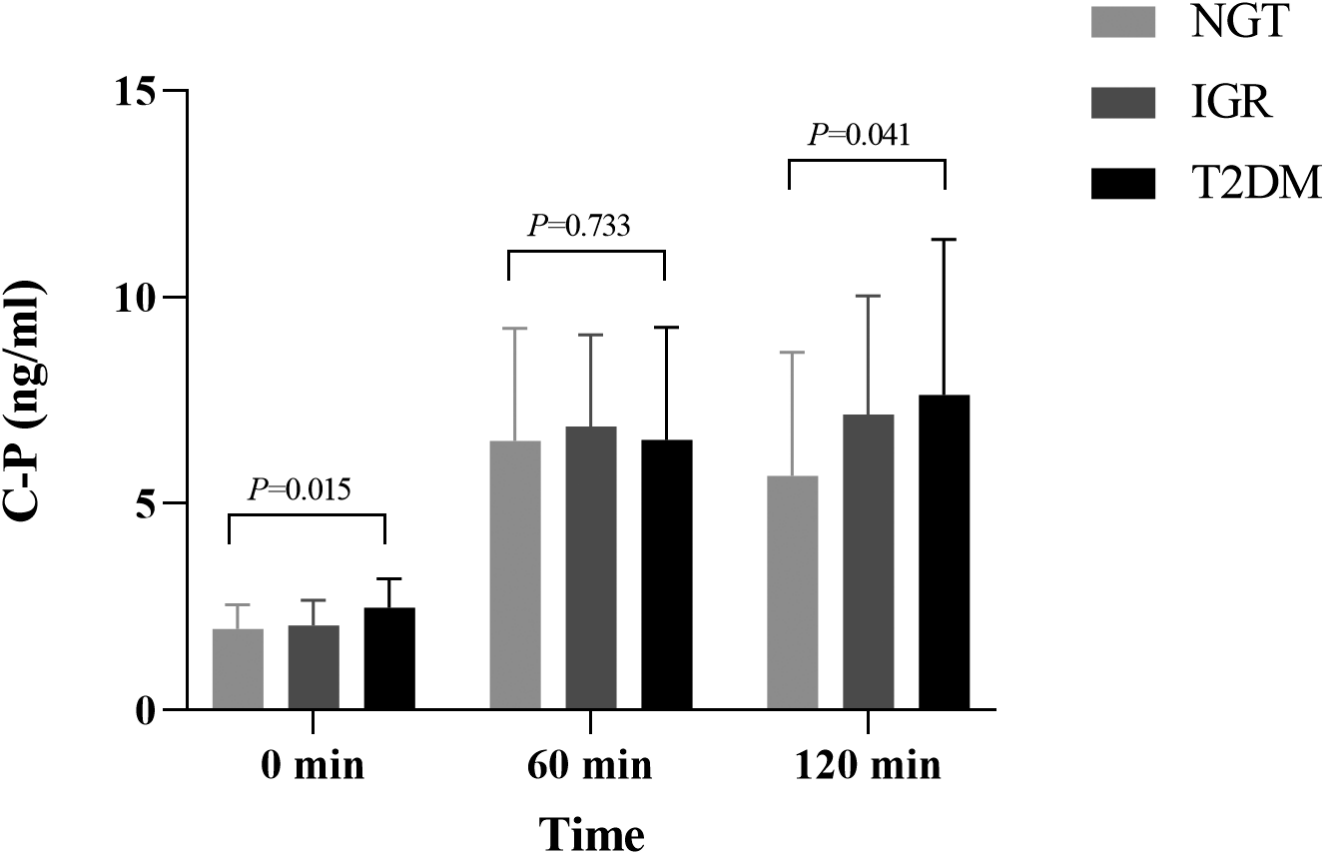
Comparison of C‐P between different glucose tolerance groups. Multiple comparisons among groups showed that the fasting C‐P in the newly diagnosed T2DM group was significantly higher than that in the NGT group (0.52101 ng·h/mL, 95% CI 0.1554–0.8867, *p* = 0.006) and the IGR group (0.42595 ng·h/mL, 95% CI 0.0632– 0.7887, *p* = 0.022), but there was no significant difference between the IGR group and the NGT group (*p* > 0.05). At 60 min after OGTT, C‐P in the newly diagnosed T2DM group was lower than that in the NGT and IGR groups, but the difference was not significant (*p* > 0.05). At 120 min after OGTT, C‐P was in the newly diagnosed T2DM group > IGR group > NGT group, and the difference between the T2DM group and the NGT group was significant (1.954265 ng·h/mL, 95% CI 0.0869–3.8216, *p* = 0.0 41), but the difference between the T2DM and IGR groups and the IGR and NGT groups did not reach significance (*p* > 0.05). CI, confidence interval; C‐P, C‐peptide; IGR, impaired glucose regulation; NGT, normal glucose tolerance; OGTT, oral glucose tolerance test; T2DM, type 2 diabetes mellitus

The general clinical characteristics and biochemical indexes of the study population in NGT, IGR, and newly diagnosed T2DM groups are shown in Table 1, including the trend comparison of GC, INS, and C‐P between the groups (Figures 1, 2, 3).

**TABLE 1:** Comparison of clinical data with different glucose tolerance groups (*x*¯±s)

Clinical features	NGT groups (37)	IGR groups (34)	T2DM groups (29)	*p* value
Age (years)	56.54 ± 8.13^c^	59.03 ± 9.50	61.83 ± 8.01^a^	0.052
Gender (female%)	25 (67.6%)	25 (73.5%)	18 (62.1%)	0.622
BMI (kg/m^2^)	25.22 ± 2.85^c^	25.00 ± 3.08^c^	26.93 ± 2.43^a^ ^,^ ^b^	0.016
Waist circumference (cm)	86.46 ± 6.60	86.97 ± 6.93	90.42 ± 5.50	0.044
SBP (mm Hg)	146.89 ± 20.76	148.29 ± 15.77	152.17 ± 24.18	0.567
DBP (mm Hg)	85.22 ± 12.03	85.76 ± 10.00	86.41 ± 12.61	0.916
HbA1c (%)	5.53 ± 0.31^b^ ^,^ ^c^	5.91 ± 0.42^ac^	6.88 ± 1.05^a^ ^,^ ^b^	<0.001
FBG (mmol/L)	5.17 ± 0.50	6.06 ± 0.63	7.39 ± 1.90	<0.001
LDL‐c (mmol/L)	3.17 ± 0.76	3.46 ± 0.64	3.36 ± 0.77	0.240
HDL‐c (mmol/L)	1.56 ± 0.31	1.56 ± 0.29	1.42 ± 0.38	0.146
TC (mmol/L)	5.60 ± 0.89	5.83 ± 0.95	5.74 ± 1.11	0.585
TG (mmol/L)	1.34 ± 0.74	1.35 ± 0.72	1.47 ± 0.55	0.713
FFA (mmol/L)	0.44 ± 0.18	0.43 ± 0.20	0.50 ± 0.21	0.354
SUA (mmol/L)	347.08 ± 75.83	326.29 ± 86.51	344.66 ± 85.64	0.525
Creatinine (umol/L)	67.10 ± 18.93	69.85 ± 19.96	72.55 ± 22.48	0.558
BUN (mmol/L)	5.29 ± 1.68	5.98 ± 7.17	5.83 ± 1.93	0.791
ALT (U/L)	26.31 ± 18.70	21.85 ± 8.82	24.40 ± 17.48	0.488
AST (U/L)	23.37 ± 11.34	20.40 ± 5.93	20.56 ± 7.07	0.272
HOMA‐IR	2.09 ± 0.85^c^	2.91 ± 1.38	4.47 ± 2.89^a^	<0.001
HOMA‐β	111.09 ± 42.16^b^ ^,^ ^c^	81.91 ± 35.13^a^	90.25 ± 64.92^a^	0.010
AUC_cp_ (ng·h/mL)	10.77 ± 4.28	11.37 ± 3.69	11.68 ± 4.83	0.791
AUC_ins_ (uIU·h/mL)	80.62 ± 71.71	94.07 ± 56.35	101.21 ± 73.24	0.615
AUC_pp_ (pg·h/mL)	4552.38 ± 874.26	4981.13 ± 621.31	4956.72 ± 807.70	0.052
AUC_gc_ (pg·h/mL)	622.56 ± 99.72^c^	658.70 ± 67.10	672.24 ± 106.50^a^	0.125
PP_0_	1603.65 ± 538.46	1687.26 ± 421.90	1818.68 ± 503.18	0.225
PP_60_	2663.55 ± 594.89^b^	2973.80 ± 547.49^a^	2828.93 ± 616.24	0.108
PP_120_	2179.79 ± 583.32	2330.07 ± 455.94	2392.51 ± 456.17	0.218
AUC_pp_	4552.38 ± 874.26^b^ ^,^ ^c^	4981.13 ± 621.31^a^	4956.72 ± 807.70^a^	0.052
GC_0_	220.93 ± 64.12	240.97 ± 69.74	254.97 ± 75.22	0.157
GC_60_	350.84 ± 79.53	375.19 ± 62.10	376.97 ± 85.69	0.311
GC_120_	318.72 ± 59.33	325.49 ± 60.96	328.10 ± 62.82	0.824
AUC_gc_	622.56 ± 99.72^c^	658.70 ± 67.10	672.24 ± 106.50^a^	0.125
INS_0_	8.98 ± 3.36^c^	10.58 ± 4.73^c^	14.20 ± 6.06^a^ ^,^ ^b^	0.001
INS_60_	51.34 ± 45.64	62.80 ± 36.40	62.43 ± 46.47	0.536
INS_120_	36.77 ± 40.96	58.83 ± 40.74	62.56 ± 54.29	0.094
AUC_ins_	80.62 ± 71.71^c^	94.07 ± 56.35^c^	101.21 ± 73.24^a^ ^,^ ^b^	0.615
C‐P_0_	1.96 ± 0.60^c^	2.05 ± 0.61^c^	2.48 ± 0.70^a^ ^,^ ^b^	0.015
C‐P_60_	6.51 ± 2.73	6.86 ± 2.23	6.54 ± 2.73	0.861
C‐P_120_	5.67 ± 3.00^c^	7.16 ± 2.87	7.63 ± 3.76^a^	0.085

^a^ *p* < 0.05, compared with NGT group.

^b^ *p* < 0.05, compared with IGR group.

^c^ *p* < 0.05, compared with T2DM group.

Abbreviations: ALT, alanine transaminase; AST, aspartate transaminase; AUC_cp_, AUC_gc_, AUC_ins_, AUC_pp_, area under the curve for C‐peptide, glucagon, insulin, pancreatic polypeptide; BMI, body mass index; BUN, blood urea nitrogen; C‐P_0_, C‐P_60_, C‐P_120_, C‐peptide at fasting, 60, and 120 min; DBP, diastolic blood pressure; FBG, fasting blood glucose; FFA, free fatty acid; GC_0_, GC_60_, GC_120_, glucagon at fasting, 60, and 120 min; HbA1C, glycosylated hemoglobin; HDL‐c, high‐density lipoprotein cholesterol; HOMA‐β, homeostatic model assessment of beta cell function; HOMA‐IR, homeostatic model assessment of insulin resistance; IGR, impaired glucose regulation; INS_0_, INS_60_, INS_120_, insulin at fasting, 60, and 120 min; LDL‐c, low‐density lipoprotein cholesterol; NGT, normal glucose tolerance; PP_0_, PP_60_, PP_120_, pancreatic polypeptide at fasting, 60, and 120 min; SBP, systolic blood pressure; SUA, serum uric acid; T2DM, type 2 diabetes mellitus; TC, total cholesterol; TG, triglyceride.

**FIGURE 4:**
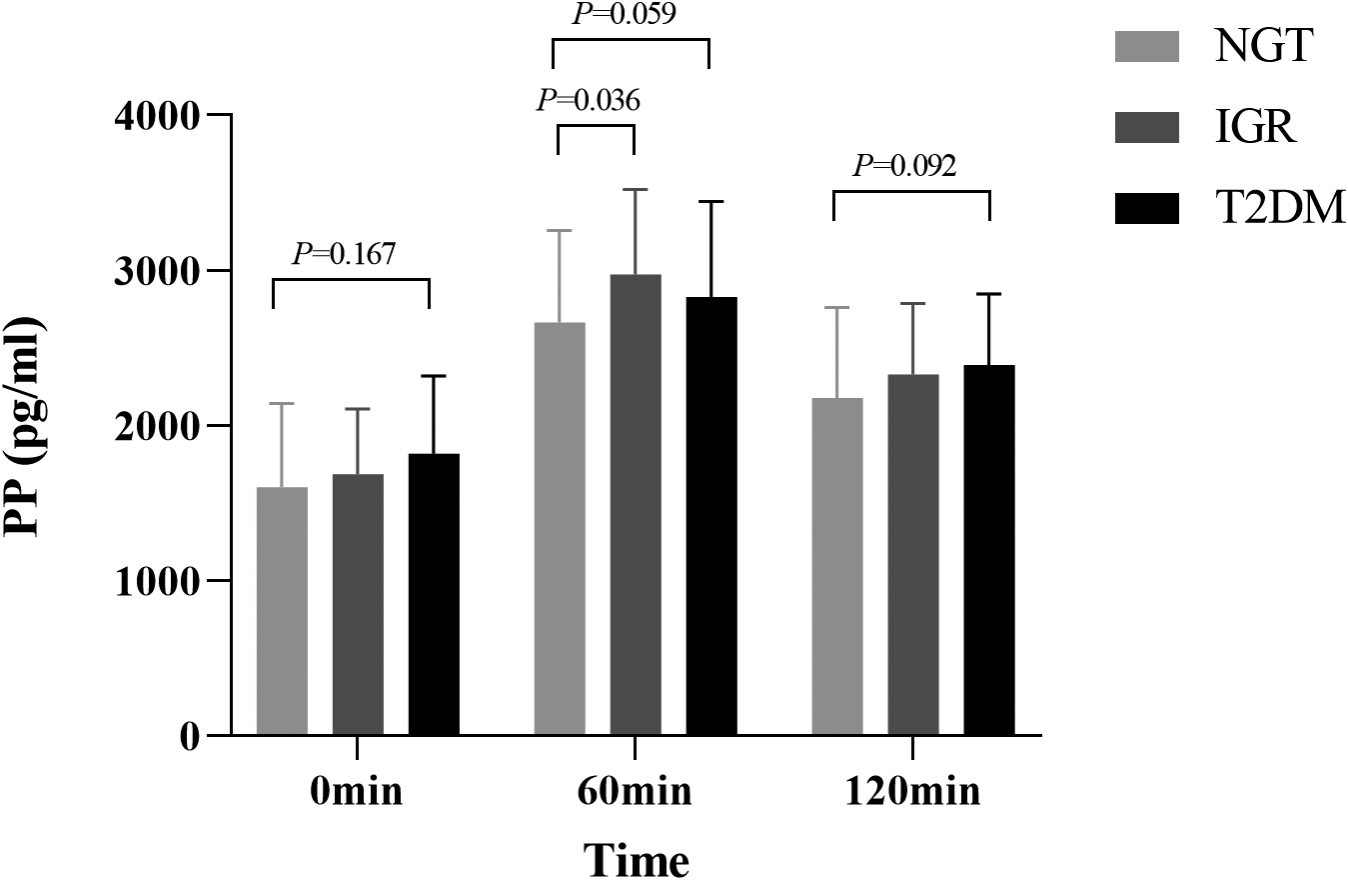
Comparison of PP between different glucose tolerance groups. After the oral administration of 75 g glucose, the concentration of PP gradually increased and reached the peak of secretion 60 min after the administration of glucose, which was about 1.5 ~ 2 times of the basal level. After that, the level of PP gradually decreased and dropped to the basal level 120 min after the administration of glucose. Multiple comparison between groups showed that fasting PP was in the newly diagnosed T2DM group > IGR group > NGT group, and there was no significant difference between groups (*p* > 0.05); PP at 60 min after OGTT was in the IGR group > T2DM group > NGT group, and the difference between IGR group and NGT group was significant (310.24 pg·h/mL, 95% confidence interval 21.05–599.44, *p* = 0.036); PP at 120 min after OGTT was in the newly diagnosed T2DM group > IGR group > NGT group, and there was no significant difference between groups (*p* > 0.05). IGR, impaired glucose regulation; NGT, normal glucose tolerance; OGTT, oral glucose tolerance test; PP, pancreatic polypeptide; T2DM, type 2 diabetes mellitus

The comparison of PP trend between different glucose tolerance groups is shown in Table 1 (Figure 4). The difference in PP at different time points was statistically significant (*p* < 0.001), and the differences in interaction between different glucose tolerance groups, between groups, and time did not reach statistical significance (*p* > 0.05). After the oral administration of 75 g glucose, the concentration of PP gradually increased and reached the peak of secretion 60 min after the administration of glucose, which was about 1.5 to 2 times of the basal level. After that, the level of PP gradually decreased and dropped to the basal level 120 min after the administration of glucose. Multiple comparison between groups showed that fasting PP was in the newly diagnosed T2DM group > IGR group > NGT group, and there was no significant difference between groups (*p* > 0.05); PP at 60 min after OGTT was in IGR group > T2DM group > NGT group, and the difference between IGR group and NGT group was significant (2973.80 [±547.49] pg·h/mL vs 2663.55 [±594.89] pg·h/mL, *p* < 0.05); PP at 120 min after OGTT was in the newly diagnosed T2DM group > IGR group > NGT group, and there was no significant difference between groups (*p* > 0.05). AUC_pp_ in the newly diagnosed T2DM group and IGR group was higher than that in the NGT group; the differences were significant (404.35 pg·h/mL, 95% CI 5.37–803.33, *p* = 0.047; 428.76 pg·h/mL, 95% CI 41.06–816.46, *p* = 0.031), but there was no significant difference between the newly diagnosed T2DM group and the IGR group (*p* > 0.05). These results suggested that the secretion of PP varies greatly at different time points of OGTT, and PP secretion was increased under abnormal glucose metabolism, but the change trend over time was not markedly affected by glucose metabolism status.

As seen in Table 2, the results showed that fasting PP was positively correlated with the course of diabetes and AUC_bg_ (r = 0.256, r = 0.243, r = 0.257, *p* < 0.05), and was negatively correlated with 60 min postprandial C‐P, 60 min postprandial insulin, and fasting GLP‐1 (r = −0.248, r = −0.235, r = −0.207, *p* < 0.05). At 60 min after OGTT, PP was positively correlated with 60 min postprandial BG (r = 0.302, *p* < 0.05). At 120 min after OGTT, PP was negatively correlated with UACR (r = −0.315, *p* < 0.05). AUC_pp_ was positively correlated with 60 min postprandial BG and AUC_bg_ (r = 0.370, r = 0.323, *p* < 0.05) and negatively correlated with BMI (r = −0.235, *p* < 0.05). The basis of PP and the secretion after glucose stimulation were not significantly correlated with the subject's age, waist circumference, SBP, DBP, HbA1c, FBG, 120 min postprandial BG, fasting C‐P, 120 min postprandial C‐P, fasting INS, 120 min postprandial INS, GC, LDL‐c, HDL‐c, TC, TG, FFA, UA, BUN, estimated glomerular filtration rate, ALT, AST, HOMA‐IR, HOMA‐β, AUC_c−p_, AUC_gc_, and AUC_ins_.

**TABLE 2:** Analysis of related factors of PP in type 2 diabetic patients

Clinical features	PP_0_		PP_60_		PP_120_		AUC_pp_	
	r	*P*	r	*P*	r	*P*	r	*P*
Age (years)	−0.04	0.7	0.017	0.872	−0.034	0.737	−0.046	0.664
Diabetes course (years)	0.256	0.011*	0.002	0.984	0.060	0.555	0.109	0.305
BMI (kg/m^2^)	−0.020	0.844	−0.140	0.180	−0.091	0.372	−0.235	0.038
Waist circumference (cm)	0.093	0.373	−0.041	0.701	0.142	0.168	0.049	0.651
SBP (mm Hg)	−0.092	0.37	−0.041	0.697	−0.134	0.187	−0.094	0.377
DBP (mm Hg)	−0.144	0.159	0.003	0.976	0.055	0.590	−0.006	0.953
HbA1c (%)	0.112	0.276	0.119	0.258	0.148	0.145	0.159	0.132
FBG (mmol/L)	0.182	0.074	0.030	0.779	0.178	0.078	0.125	0.237
BG_60_ (mmol/L)	0.243	0.033*	0.302	0.008**	0.148	0.196	0.370	0.001**
BG_120_ (mmol/L)	0.110	0.388	0.097	0.450	0.050	0.695	0.171	0.183
C‐P_0_ (ng·h/mL)	−0.039	0.744	0.076	0.526	0.057	0.626	0.074	0.544
C‐P_60_ (ng·h/mL)	−0.248	0.032*	−0.099	0.399	−0.051	0.660	−0.159	0.177
C‐P_120_ (ng·h/mL)	−0.185	0.117	0.074	0.534	−0.187	0.110	−0.057	0.632
INS_0_ (uIU·h/mL)	0.058	0.626	0.113	0.350	0.059	0.614	0.148	0.222
INS_60_ (uIU·h/mL)	−0.235	0.042*	−0.109	0.354	0.001	0.995	−0.152	0.197
INS_120_ (uIU·h/mL)	−0.187	0.114	0.019	0.872	−0.100	0.396	−0.077	0.522
Fasting GLP‐1 (pmol/L)	−0.207	0.048*	−0.142	0.192	0.010	0.928	−0.176	0.105
GC_0_ (pg·h/mL)	0.058	0.576	0.054	0.620	0.166	0.110	0.115	0.284
GC_60_ (pg·h/mL)	0.009	0.931	−0.025	0.816	0.110	0.297	0.009	0.932
GC_120_ (pg·h/mL)	−0.170	0.112	−0.048	0.661	0.020	0.850	−0.085	0.437
LDL‐c (mmol/L)	0.063	0.542	0.037	0.728	−0.070	0.488	0.020	0.850
HDL‐c (mmol/L)	−0.082	0.426	0.092	0.378	−0.117	0.247	−0.012	0.909
TC (mmol/L)	0.0005	0.960	0.040	0.707	−0.101	0.321	−0.024	0.822
TG (mmol/L)	−0.014	0.893	0.029	0.782	−0.031	0.763	0.029	0.782
FFA (mmol/L)	−0.189	0.072	−0.134	0.215	−0.107	0.308	−0.163	0.133
UACR (mmol/L)	0.103	0.496	−0.078	0.615	−0.315	0.033*	−0.131	0.402
SUA (mmol/L)	0.007	0.948	0.118	0.262	0.085	0.401	0.137	0.197
BUN (mmol/L)	−0.004	0.971	0.093	0.373	−0.062	0.545	0.049	0.647
ALT (U/L)	0.055	0.595	−0.094	0.372	0.005	0.959	−0.063	0.552
AST (U/L)	−0.084	0.415	−0.098	0.352	−0.018	0.858	−0.109	0.303
HOMA‐IR	0.152	0.188	0.081	0.494	0.078	0.496	0.143	0.227
HOMA‐β	−0.144	0.210	0.037	0.755	−0.046	0.692	−0.008	0.950
AUC_bg_ (mmol/L)	0.257	0.050*	0.247	0.059	0.068	0.604	0.323	0.007*
AUC_cp_(ng·h/mL)	−0.156	0.231	0.029	0.824	−0.067	0.604	−0.046	0.726
AUC_ins_(uIU·h/mL)	−0.108	0.405	0.013	0.922	−0.098	0.446	−0.048	0.709
AUC_gc_(pg·h/mL)	−0.007	0.947	−0.034	0.758	0.136	0.208	0.024	0.825

*Note*: *p < 0.05, **p < 0.01.

Abbreviations: ALT, alanine transaminase; AST, aspartate transaminase; AUC_bg_, AUC_cp_, AUC_gc_, AUC_ins_, AUC_pp_, area under the curve for blood glucose; BG_60_, BG_120_, blood glucose at 60 and 120 min; C‐peptide, glucagon, insulin, pancreatic polypeptide; BMI, body mass index; BUN, blood urea nitrogen; C‐P_0_, C‐P_60_, C‐P_120_, C‐peptide at fasting, 60, and 120 min; DBP, diastolic blood pressure; FBG, fasting blood glucose; FFA, free fatty acid; GC_0_, GC_60_, GC_120_, glucagon at fasting, 60, and 120 min; GLP‐1, glucagon‐like peptide‐1; HbA1C, glycosylated hemoglobin; HDL‐c, high‐density lipoprotein cholesterol; HOMA‐β, homeostatic model assessment of beta cell function; HOMA‐IR, homeostatic model assessment of insulin resistance; INS_0_, INS_60_, INS_120_, insulin at fasting, 60, and 120 min; LDL‐c, low‐density lipoprotein cholesterol; PP_0_, PP_60_, PP_120_, pancreatic polypeptide at fasting, 60, and 120 min; SBP, systolic blood pressure; SUA, serum uric acid; TC, total cholesterol; TG, triglyceride; UACR, urine albumin‐creatinine ratio.

AUC_pp_ was used as the dependent variable and the potentially influencing factors such as BMI and AUC_bg_ were used as the independent variable for stepwise fitting. After adjusting for TC, TG, LDL‐C, HDL‐C, ALT, AST, SBP, DBP, and age, multiple linear regression analysis showed that there was a linear correlation between BMI, AUC_bg_, and AUC_pp_ (*p =* 0.011, *p =* 0.001), and the regression equation was calculated as AUC_pp_ = 6592.272 + 86.275 × AUC_bg_‐95.291 × BMI (R^2^ = 12.7%, *p* < 0.05).

## 4 DISCUSSION

This study explored the differences of PP in different glucose metabolism states and analyzed the influencing factors of PP secretion by measuring the serum concentration of PP, GC, INS, and C‐P in people with different glucose tolerance states before and after glucose loading. The results showed that, compared with NGT, the postprandial secretory response of PP to oral glucose stimulation was enhanced in IGR (including IFG and IGT) and newly diagnosed T2DM subjects. The secretion of PP was mainly affected by BMI and BG level, which was negatively correlated with BMI and positively correlated with BG level. We hypothesized that the disorder of PP secretion after glucose stimulation in people with abnormal glucose metabolism might be related to the disorder of autonomic (parasympathetic) nerve system, and the specific mechanism remains to be further studied in the future.

PP is secreted mainly by pancreatic PP cells,^23^ which form the endogenous ligand of hypothalamic Y4 receptor (also known as PPYR4). Previous studies have shown that the secretion of PP is vagus‐cholinergic dependent and could be inhibited by anticholinergic drugs or blocked by vagal nerve transection.^24^ In addition to being mainly affected by cholinergic stimulation and feeding factors, gastric fundus dilatation can also reflexively cause an increase in circulating PP, which disappeared after selective removal of innervation in the gastric fundus area.^25^ Intraduodenal infusion of bile and pancreatic juice also stimulates the secretion of PP.^26^ In addition, in the early stage, Sive et al^27^ found that the co‐perfusion of adrenaline with α‐receptor blockers such as phentolamine could significantly increase the release of serum PP, suggesting that the sympathetic nerve was also involved in the regulation of PP secretion.

In this study, we found an enhanced secretion response of PP to oral glucose stimulation in patients with IGR and T2DM compared with those with NGT. This result is consistent with previous findings that diabetes patients with poor glycemic control have enhanced PP reactive secretion.^28^, ^29^, ^30^ Berger et al^28^ and Skare et al^31^ found that the normalization of fasting BG in T2DM patients through lifestyle intervention and insulin therapy was associated with a decrease of basal and secretory activity of PP cells. Floyd et al^9^ also observed an increase in plasma PP in diabetic patients, which seems to be correlated with the severity of hyperglycemic states. Besides, it has been further observed that oral glucose load could cause an overreaction of PP secretion in untreated diabetic patients.^32^ Consistently, the analysis of PP influencing factors in this study also showed that PP secretion after OGTT was positively correlated with BG concentration. We speculated that this phenomenon might be correlated with the disorder of autonomic (parasympathetic) nerve function under the condition of abnormal glucose metabolism in the IGR and T2DM population. Such hyperactive PP secretion may be secondary to the overactive condition of the vagus nerve in those people. However, the underlying mechanism of this finding may be far more complex. Contrary to the results of this study, Tasaka et al^33^ measured the content of PP in the pancreas of 24 autopsied diabetic and 19 nondiabetic people and found that there was no correlation between the content of pancreatic PP and the stability of FBG. We speculated that it may be due to the different source of PP samples. Tasaka et al^33^ used acid alcohol to extract tissue after mincing the pancreas to measure PP content, whereas this study measured PP content in serum. In addition, there is evidence that hypoglycemia is a strong stimulator of PP secretion,^3^, ^34^, ^35^, ^36^, ^37^ and the degree of PP secretion is directly proportional to the degree of hypoglycemia, which is regulated by the vagus nerve,^38^, ^39^ whereas hyperglycemia can inhibit PP secretion.^38^, ^39^, ^40^ On the other hand, the elevated PP level may also be related to the proliferation of pancreatic PP cells in IGR and T2DM population. Previously, researchers observed proliferation of PP cells in diabetic patients with poor BG control,^29^, ^30^ which may be the result of atypical islet regeneration caused by severe or long‐term damage of pancreatic endocrine tissue. However, how the number of PP cells changes in patients with T1DM or T2DM is still controversial, and there is no consensus as to whether the number of PP cells increases^29^, ^30^, ^41^ or is unchanged.^27^, ^42^ In addition, this study also found that the increase of PP in IGR patients after oral glucose stimulation was more obvious than that in T2DM patients. We speculated that this result might be related to diabetic autonomic neuropathy in T2DM patients, such as sympathetic hyperfunction and decreased vagal function, which would lead to impaired reactive secretion of PP. Studies have shown that diabetic patients have impaired PP secretion response to hypoglycemia and eating stimulation, especially in people with diabetic autonomic neuropathy,^43^, ^44^ which further supports our hypothesis. Therefore, in this study, we observed a relative decrease in the secretion of postprandial PP stimulation in T2DM patients compared with IGR patients, which may be related to the disorder of the vagus nerve system related to the diabetic autonomic neuropathy in T2DM patients.

In this study, the correlation factor analysis of PP showed that there was a significant negative correlation between C‐P and PP concentration in basal conditions. Previously, Weyer et al^45^ found significant hyperinsulinemia and elevated PP concentrations in the Pima Indian population at high risk of T2DM. Kahleova et al^46^ also indicated that INS secretion was negatively correlated with PP secretion in T2DM patients with hyperinsulinemia. They also found that after weight loss induced by lifestyle intervention, PP secretion decreased with the improvement of islet β‐cell function.^46^ Study showed that the functional defects of β‐cells in patients with chronic pancreatitis are related to the damage of PP cells.^19^, ^20^, ^21^ PP infusion can reverse the abnormal glucose metabolism after pancreatectomy,^22^ suggesting that β‐cells located in the “center” of the pancreatic islet are protected from destruction by surrounding PP cells, and the reduction of PP may be the first signal of pancreatic islet cell dysfunction. In this study, the positive correlation (*p* > 0.05) between C‐P and PP concentration at 60 min after glucose load is further consistent with this point.

The development of diabetes is not only because of the secretory dysfunction of pancreatic endocrine cells but also the structural change of pancreatic islet and the disorder of communication signals between pancreatic endocrine cells.^47^ In recent years, the main subtypes of neuropeptide Y receptor (NPYR) including NPYR1, 2, 4, and 5 have been found to be expressed in mouse islets,^17^ and PP has a strong affinity with NPYR4, suggesting that PP may have potential paracrine interactions with pancreatic endocrine cells.^18^ There is a suggestion that activation of NPYRs by PP could protect against β‐cell loss by preventing cell apoptosis rather than having direct proliferative effects.^48^, ^49^ Taken together, we speculate that PP may have a potential protective effect on the survival and function of β‐cells.

The correlation factor analysis of PP in this study showed that there was a significant negative correlation between GLP‐1 and PP in the basal state. Recent studies have shown that exogenous GLP‐1 administration significantly weakens the effects of postprandial INS, GC, and PP secretion after vagal nerve transection.^50^ It has been suggested that GLP‐1 may reduce the release of PP by inhibiting cholinergic activity in the efferent vagus nerve and may cause delayed gastric emptying through at least one central nervous pathway.^51^ In addition, GLP‐1 can stimulate INS secretion and regulate GC secretion in a glucose‐dependent manner,^52^, ^53^, ^54^ which may also indirectly affect PP secretion.

In conclusion, compared with NGT subjects, IGR and T2DM patients have an enhanced postprandial PP secretion, which tends to be more significant in IGR subjects. In T2DMs, the secretion of PP is mainly affected by BMI and BG. There may exist a potential protective effect of PP on the function of islet cells. Although further prospective cohort studies and mechanism exploration are still needed to figure out the causal relationship in the future, our findings provide novel insights into the potential roles of PP in the pathogenesis of established diabetes.

## DISCLOSURE

The authors declare no conflict of interest.
